# Dioxins levels in human blood after implementation of measures against dioxin exposure in Japan

**DOI:** 10.1186/s12199-018-0755-7

**Published:** 2019-01-10

**Authors:** Basilua Andre Muzembo, Miyuki Iwai-shimada, Tomohiko Isobe, Kokichi Arisawa, Masayuki Shima, Tetsuhito Fukushima, Shoji F. Nakayama

**Affiliations:** 1 Centre for Health and Environmental Risk Research, National Institute for Environmental Studies, Tsukuba, 305-8506 Japan; 2 Institute of Biomedical Sciences, Tokushima University Graduate School, Tokushima, 770-8503 Japan; 3 Department of Public Health, Hyogo College of Medicine, Nishinomiya, 663-8501 Japan; 4 Department of Hygiene and Preventive Medicine, School of Medicine, Fukushima Medical University, Fukushima, 960-1295 Japan; 5 Department of Public Health, School of Medicine, International University of Health and Welfare, Narita, 286-8686 Japan

**Keywords:** Coplanar polychlorinated biphenyls, Cross-sectional sample, Dietary dioxin intake, Dioxins, Polychlorinated dibenzo-p-dioxins, Polychlorinated dibenzofurans

## Abstract

**Background:**

Over the past few decades, the Japanese Ministry of the Environment has been biomonitoring dioxins in the general Japanese population and, in response to public concerns, has taken measures to reduce dioxin exposure. The objectives of this study were to assess the current dioxin dietary intake and corresponding body burden in the Japanese and compare Japanese dioxin data from 2011 to 2016 and 2002–2010 surveys. We also examined the relationship between blood dioxins and health parameters/clinical biomarkers.

**Methods:**

From 2011 to 2016, cross-sectional dioxin surveys were conducted on 490 Japanese (242 males and 248 females, aged 49.9 ± 7.6 years) from 15 Japanese prefectures. Blood (*n* = 490) and food samples (*n* = 90) were measured for 29 dioxin congeners including polychlorinated dibenzo-para-dioxins (PCDDs), polychlorinated dibenzofurans (PCDFs), and coplanar polychlorinated biphenyls (Co-PCBs) using gas chromatography coupled with high-resolution mass spectrometry. Using the 2006 World Health Organization toxic equivalence factors, the toxic equivalents (TEQs) were calculated. Clinical biomarkers and anthropometric parameters were also measured and information on lifestyle behaviours collected. Data imputations were applied to account for blood dioxins below the detection limit.

**Results:**

The median (95% confidence interval or CI) blood levels and dioxin dietary intake was respectively 9.4 (8.8–9.9) pg TEQ/g lipid and 0.3 (0.2–0.4) pg TEQ/kg body weight/day. The median blood dioxin level in the 2011–2016 survey was found to have decreased by 41.3% compared to the 2002–2010 surveys. Participants who were older were found to be more likely to have higher dioxin levels. Blood dioxins were also significantly associated with body mass index, triglycerides, docosahexaenoic acid, eicosapentaenoic acid, and dihomo-gamma-linoleic acid levels in blood. Furthermore, associations between blood dioxin and dietary dioxin intake were evident in the unadjusted models. However, after adjusting for confounders, blood dioxins were not found to be associated with dietary dioxin intake.

**Conclusions:**

Blood dioxin levels declined over the past decade. This study showed that the measures and actions undertaken in Japan have possibly contributed to these reductions in the body burden of dioxins in the Japanese population.

**Electronic supplementary material:**

The online version of this article (10.1186/s12199-018-0755-7) contains supplementary material, which is available to authorized users.

## Introduction

Human exposure to persistent organic pollutants (POPs) such as dioxins has become a public health concern as these have been linked to serious health conditions such as cancer [1], diabetes and hypertension [2, 3]. The generic term 'dioxins' refers to polychlorinated dibenzo-para-dioxins (PCDDs), polychlorinated dibenzofurans (PCDFs) and dioxin-like coplanar polychlorinated biphenyls (Co-PCBs), all of which are by-products of waste incineration and chemical manufacturing processes [4]. Because of their environmental persistence and lipophilicity, and the capacity to be carried long distances in the air before being deposited in water and soils, these environmental pollutants bio-accumulate in food and human bodies [5–7].

Most human dioxin exposure in Japan is because of food consumption and especially from the dioxins in fish and sea food [8]. Mass poisonings in Japan have been caused by dioxin-contaminated rice oil, which was called the Yusho oil disease [9], and from 1990 to 1997, elevated levels of dioxins from municipal solid waste incineration plants contaminated the soil surrounding the incinerator plants [7, 10].

Recognising the public’s concerns with the high level of dioxin pollution, the Japanese government promulgated regulations and implemented preventive measures to reduce dioxin emissions from flue gases and sought to prevent the de novo formation of dioxins. These measures included a dioxins law related to ‘special measures against dioxins’ released in 1999 [11], improvements in incineration facilities, adequate waste management and recycling, the clean-up of soil contaminated with dioxins, and the detoxification and decomposition of dioxins [7, 11, 12], with most of these nationwide counter-measures being put in place by 2004. The Ministry of the Environment of Japan (MOEJ) reported that these regulatory efforts had resulted in a 95% decline in emitted dioxins (341 g TEQ; toxic equivalent) by 2004 and a further 98% reduction by 2010 (158–160 g TEQ) [12] compared to 1997 dioxin emissions (7680 g TEQ) [13].

To further evaluate the effectiveness of its actions, the MOEJ has been biomonitoring dioxins in non-occupationally exposed individuals. The main problem with dioxins is that they are lipophilic and can easily enter the food chain and, as they are resistant to degradation, remain in the environment. Because the public was concerned about the heightened dioxin levels found in Japan, the biomonitoring of dioxins and other chemicals was undertaken to evaluate the exposure and to prevent potentially adverse effects. From 2002 to 2010, the MOEJ conducted ‘the survey on the exposure to dioxins and other chemical compounds (including pesticides and plasticizers) in humans’ (SEDOCCH) [2, 3, 8, 14–20] nationwide (SEDOCCH 2002–2010). The latter aimed to clarify the relationship between the dioxin dietary intake and corresponding body burden and other POPs in the Japanese general population. The results revealed that the dioxin dietary intake and corresponding TEQ dioxins in the blood (median = 16 pg TEQ/g lipid) were associated with adverse health effects such as diabetes [2]. SEDOCCH 2002–2010 also found that blood and food dioxins had had an almost twofold decrease between 2002 and 2010 and provided evidence-based data for MOEJ and researcher dioxins surveillance evaluations. Because of the public concern, the dioxins biomonitoring project was continued every year from 2011 to 2016 (SEDOCCH 2011–2016).

The objectives of this study were to assess current blood dioxin levels and dietary intake of dioxins in Japanese from the 2011–2016 SEDOCCH data. We also sought to examine the relationship between blood dioxins and health parameters/clinical biomarkers. In addition, data from the dioxins biomonitoring conducted from 2011 and 2016 (SEDOCCH 2011–2016) were compared to the results in the 2002–2010 SEDOCCH.

## Methods

### Study population

A dataset sample from the MOEJ cross-sectional 2011 to 2016 SEDOCCH was used. Sampling from 2011 to 2016 primarily focused on the 15 prefectures in which the dioxin levels had been found to be high (median blood dioxins ranging from 21.5 to 40.9 pg TEQ/g lipid) or low (median blood dioxins between 8.3 and 14.1 pg TEQ/g lipid) in the 2002–2010 SEDOCCH surveys [16]. The participants were selected from the urban areas, agricultural/farming areas and fishing villages, which were defined as in previous reports [2, 3]. Eighty participants were planned to be recruited each survey year through invitation by local administrative authority using public relations magazines/pamphlets. The inclusion criteria were as follows: adult aged ≥ 20, no known occupational exposure to dioxins, living in the study area for at least 10 years, rarely absent from the survey region for work or other reasons, and no medical restrictions for providing blood samples. Pregnant women and people with diseases, such as anaemia, were excluded because of possible interferences with the blood sampling or dioxin measurement. After consent was obtained, the eligible participants were invited for an interviewer-administered questionnaire, anthropometry evaluations [blood pressure, height, weight and body mass index (BMI)] and dioxin exposure assessments. In each survey year, 15 (18.8%) individuals among the participants were randomly chosen to provide duplicate diet samples.

### Questionnaire

This study used the same health, lifestyle and food frequency questionnaires as used in the 2002–2010 SEDOCCH survey [16, 19, 20]. Briefly, a trained public health nurse or nutritionist administered the questionnaire face-to-face. The questionnaire had been sent to participants in advance for completion at home, after which the participants were invited to the local community centres for the interview. The core questionnaire gathered sociodemographic information, current health status and personal medical history (including medication and diseases diagnoses such as cancer, diabetes, hypertension and hyperlipidaemia, residential history, occupational history, tobacco use and alcohol consumption). A semi-structured and open-ended question food frequency questionnaire was also used to collect information on food group intake frequency, in which the frequencies for 41 foods, food groups, menus and beverages during the previous month were recorded. For example, fish consumption frequency was reported in categorical format using the following eight choices: (1) Not at all, (2) 1–3 times per month, (3) 1–2 times per week, (4) 3–4 times per week, (5) 5–6 times per week, (6) once per week, (7) twice per day and (8) 3 times or greater per day.

### Blood and duplicate diet sampling

Blood drawing was performed on the same day as the interview survey. The blood collection, processing and analytical procedures were a revised version of those in the 2002–2010 SEDOCCH, which have been published elsewhere [18]. Nearly 50 ml (vs. 20 ml in the 2002–2010 SEDOCCH) of fasting venous blood was drawn by a nurse or a clinical laboratory technician under the supervision of a physician using different vacutainer tubes: Tubes for blood count and chemical analysis contained ethylenediaminetetraacetic acid disodium salt (EDTA-2NA); one for blood glucose, insulin and haemoglobin A1c (HbA1c) carried sodium fluoride, EDTA-2Na and sodium heparin, and another one for other clinical test items with coagulants. Collected blood was shipped to a contract laboratory (IDEA Consultants, Inc., Yaizu, Shizuoka, Japan).

Duplicate diet samples were primarily collected to estimate dioxin intake, and a diet sample collection was taken from a subset of the participants during the survey periods. Consecutive 3-day duplicate participants’ meals were collected by nutritionists and were kept in stainless steel containers, as previously reported by Arisawa et al. [15].

### Analyses of dioxins

The analytical dioxin methods used in this study were in accordance with the guidelines published by the Ministry of Health, Labour and Welfare as detailed in previous reports [14, 21, 22]. The blood and diet samples were weighed and homogenised before measurement. An aliquot of the sample was spiked with internal standards and a solvent extracted, which was then subjected to a multi-layer silica column and an activated carbon column chromatography for purification. Then, the PCDDs, PCDFs and Co-PCBs (non-ortho PCBs and mono-ortho-PCBs) in the blood and diet samples were quantified using gas chromatography coupled with a high-resolution mass spectrometer (IDEA Consultants, Inc.). The dietary dioxin intake in the diet samples was estimated using the following equation:

$$ \mathrm{Din}=\frac{\left(\mathrm{Ddioxin}\times \mathrm{Dsize}\right)/3}{\mathrm{BW}} $$

where Din, Ddioxin, Dsize and BW respectively denoted the dietary dioxin intake, diet dioxin concentrations (3-day duplicate diets) in pg TEQ/g, the diet sample size (total amount over 3 days) in grams, and the body weight of each subject in kilograms. Using the 2006 World Health Organization (WHO) toxic equivalence factors (TEFs), the TEQs were calculated [23]. Blood dioxins were normalised for lipids and expressed as pg TEQ/g lipid, and the diet dioxin intake was expressed as pg TEQ/kg body weight/day. The detection limits for blood dioxins (pg/g lipid) were 1, 2, 4 and 10 for PCDDs/PCDFs with four or five chlorine atoms, PCDDs/PCDFs with six or seven chlorine atoms, PCDDs/PCDFs with eight chlorine atoms, and Co-PCBs, respectively.

### Analyses of clinical biomarkers

The blood samples were also subjected to haematological and biochemical assays. Analyses of clinical biomarkers (Health Sciences Research Institute, Inc., Kanagawa, Japan) were conducted for blood glucose, HbA1c, lipids [triglycerides and high-density lipoprotein (HDL) cholesterol] and blood urea nitrogen, thyroid hormones [triiodothyronine (T3) and thyroxine (T4)] and liver function tests [aspartate aminotransferase (AST); alanine aminotransferase (ALT); gamma-glutamyl transpeptidase (GGT)]. Measurements of polyunsaturated fatty acids of eicosapentaenoic acid (EPA), docosahexaenoic acid (DHA), and dihomo-gamma-linolenic acid (DGLA) [2] were performed by SRL Inc. (Tokyo, Japan).

### Statistical methods

Stata 14 software (StataCorp LP, College Station, TX, USA) was used for the analyses. All *p* values presented were two-tailed, with *p* < 0.05 being considered significant for all statistical tests. The numerical variables were assessed for normality using the Shapiro-Wilk test, and the numerical and qualitative variables were summarised using descriptive statistics. The numerical variables were presented as the mean ± standard deviation if the distribution was normal, or as a median and interquartile range if the distribution was skewed. Categorical variables were presented as frequencies (percentages), and the differences evaluated using chi-square tests or Fisher’s exact tests. A Student’s *t* test (to compare two groups) or an ANOVA (to compare more than two groups) were used for the equal variance assessment when the data were normally distributed; however, for skewed data distributions, the Mann-Whitney *U* test (to compare two groups) or the Kruskal-Wallis test (to compare more than two groups) was used. Pearson’s correlations and multiple regression analyses were undertaken to explore the relationships between the TEQs and health parameters/clinical biomarkers. If the health parameters/clinical biomarkers correlated with the total TEQ and showed a *p* value ≤ 0.2, they were included in the multiple regression models. The models were also adjusted for smoking status and sex. As the PCDD, PCDF, Co-PCB and total dioxins (concentrations and TEQs) distributions were skewed to the right, they were presented as medians [95% confidence intervals (CI) or range]. To address the skewedness, the natural logs of dioxin congeners and total TEQ were used for the simple correlations and the multiple regression analyses. A value of zero was assigned to the PCDDs, PCDFs and Co-PCBs concentrations below the detection limit with the consideration that this could lead to bias [24]. A simple substitution of the non-detectable values was applied for comparison with the 2002–2010 SEDOCCH survey, in which a simple substitution approach (assignment of ‘zero’ to the congener concentrations below the detection limit) had been used [25]. In the 2002–2010 SEDOCCH survey, imputation of non-detects was not performed. However, in this study, to estimate the Pearson’s *r* coefficient and in the multiple regression analyses, the PCDDs, PCDFs and Co-PCBs concentrations below the limit of detection (LOD) were treated as missing data. To account for the missing dioxin data, multiple imputations by chained equations (MICE) and predictive mean matching (PMM) were applied in Stata to impute the missing data [24, 26, 27]. For missing data exceeding 10%, we used the multiple imputation strategies following the guidelines suggested by Newman [28]. Sensitivity analyses were also conducted to compare the results of the different methods used to handle the congener concentrations below the detection limit.

## Results

### Study population and their health outcome variables

**Fig. 1:**
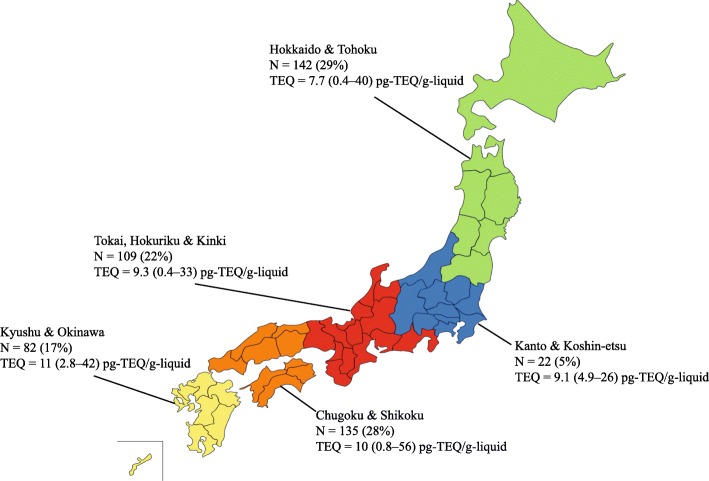
Map of Japan showing the origins of the 490 participants [*N* (%)] and their median blood dioxin total TEQ (range) in pg TEQ/g lipid. The geographic patterns of total TEQ presented in each region do not refer to region-level blood dioxin estimates. Instead, this geographic distribution represents the participants involved in this study. Definition of abbreviations: TEQ, toxic equivalent

Table 1 and Figure 1 give the details of the 490 participants [242 males (49.4%); mean age 49.9 years with a range from 24 to 77] included in the 2011–2016 SEDOCCH surveys. A total of 63.5% of participants had been recruited from prefectures in which the dioxin levels were found to be high in the 2002–2010 SEDOCCH results. The clinical and biochemical parameters differed in the male and female participants, with the BMI, smoking status, blood glucose, AST, alanine transferase ALT, GGT and serum triglycerides generally found to be higher in the males, and the serum HDL cholesterols higher in females (Table 1 and Additional file 1: Table S1). Current male smokers were more likely to have higher number of cigarettes smoked per day compared to current female smokers (19 versus 10, respectively). Furthermore, never smokers (mean age 49.9 years with a range from 36 to 72) were slightly older (*t* test, *p* = 0.047) compared with current smokers (mean age 48.5 years with a range from 24 to 63). HbA1c levels of ≥ 5.6% were found in 29% (142 of 490) of the participants indicating probably prediabetes or diabetes; however, only 7.1% self-reported a history of physician-diagnosed diabetes (Additional file 1: Table S1). The fish consumption habits were found to be high in participants from the fishing villages. For example, only 15.7% (37/235) of the participants from the fishing villages reported that they do not consume fish at all [versus 37.5% (21/56) in urban areas and 26.6% (53/199) in farming villages].

**Table 1:** Characteristics of 490 participants in SEDOCCH 2011–2016

Characteristics	Male (*N* = 242)	Female (*N* = 248)	All (*N* = 490)	*p v*alue^†^
	*n* (%) or mean (range)	*n* (%) or mean (range)	*n* (%) or mean (range)	
Age (years)^a^	49.4 (24–77)	50.3 (37–72)	49.9 (24–77)	0.135
24–39	25 (5.1)	11 (2.2)	36 (7.3)	
40–49	100 (20.4)	111 (22.6)	211 (43.1)	
50–59	98 (20.0)	98 (20.0)	196 (40.0)	
60–77	19 (3.8)	28 (5.7)	47 (9.6)	
BMI (kg/m^2^)^a^	24.5 (18.6–36.3)	23.0 (15.5–37.4)	23.7 (15.5–37.4)	< 0.001
Underweight (< 18.5)	0 (0.0)	19 (3.8)	19 (3.8)	
Normal weight (18.5–24.9)	156 (31.8)	171 (34.9)	327 (66.7)	
Overweight (25.0–29.9)	65 (13.2)	46 (9.3)	111 (22.6)	
Obese (≥ 30)	21 (4.2)	12 (2.4)	33 (6.7)	
Smoker				< 0.001
Current	86 (17.6)	16 (3.3)	102 (20.8)	
Past	94 (19.2)	23 (4.7)	117 (23.8)	
Never	61 (12.4)	207 (42.2)	268 (54.6)	
Missing	1 (0.2)	2 (0.4)	3 (0.6)	
Number of cigarettes smoked per day^a^				
Current	19 (1–40)	10 (2–20)	17 (1–40)	0.001
Past	20 (1–100)	12 (3–40)	19 (1–100)	< 0.001
Not applicable	61	207	268	
Missing	3	2	5	
Residential area				0.046
Urban	19 (3.8)	37 (7.5)	56 (11.4)	
Farming village	104 (21.2)	95 (19.3)	199 (40.6)	
Fishing village	119 (24.2)	116 (23.6)	235 (47.9)	
Year of survey				0.150
2011	51 (10.4)	35 (7.1)	86 (17.5)	
2012	35 (7.1)	49 (10.0)	84 (17.1)	
2013	38 (7.7)	45 (9.1)	83 (16.9)	
2014	35 (7.1)	46 (9.3)	81 (16.5)	
2015	39 (7.9)	37 (7.5)	76 (15.5)	
2016	44 (8.9)	36 (7.3)	80 (16.3)	

*SEDOCCH* survey on the exposure to dioxins and other chemical compounds in humans, *BMI* body mass index

^a^Mean (range)

^†^*p* values are comparing male and female participants

### Blood dioxins

The detection frequencies and selected percentiles (95% CI) for the blood dioxin congeners, expressed in pg/g lipid, and the total TEQ are listed in Table 2. Respectively, the median TEQ for the PCDDs, PCDFs and Co-PCBs were 3.8, 1.8 and 3.6 pg TEQ/g lipid, and the median total TEQ in the blood was 9.4 pg TEQ/g lipid (range, 0.39–59 pg TEQ/glipid). The highest contributions to the median total TEQ in the blood were found to come from the PCDDs (41.3%), followed by the Co-PCBs (39.1%), and the PCDFs (19.6%). When stratifying the blood dioxin levels by gender, no statistically significant differences in median blood dioxin levels across genders were found (Wilcoxon rank-sum test, *p* = 0.712). A comparison of blood dioxins by participants’ region of origin (Fig. 1) found that blood dioxin levels varied (Kruskal-Wallis test, *p* < 0.001), with the median (95% CI) TEQ being highest in participants from Kyushu/Okinawa [11.0 (9.1–12.0)] pg TEQ/g lipid, followed by those from Chugoku/Shikoku [10.0 (9.3–11.4)] pg TEQ/g lipid, Tokai/Hokuriku/Kinki [9.3 (7.8–10.0)] pg TEQ/g lipid and Kanto/Koshinetsu [9.1 (7.5–14.0)] pg TEQ/g lipid, and the lowest being found in those from Hokkaido/Tohoku [7.7 (6.5–9.3)] pg TEQ/g lipid.

**Table 2:** Blood levels of PCDDs/PCDFs/Co-PCBs (in pg/g lipid and pg TEQ/g lipid) and total TEQ of participants (*N* = 490) in SEDOCCH 2011–2016

	Frequencies of values below the LOD, *n* (%)*	25th percentile	Median	75th percentile	95th percentile
PCDDs (pg/g lipid)					
2,3,7,8-TeCDD	378 (77.1)	<LOD	<LOD	<LOD	2. 0 (2.0–2.0)
1,2,3,7,8-PeCDD	51 (10.4)	2.0 (2.0–2.0)	3.0 (3.0–3.0)	4.0 (4.0–4.0)	7.0 (6.0–8.0)
1,2,3,4,7,8-HxCDD	442 (90.2)	<LOD	<LOD	<LOD	3.0 (2.0–3.0)
1,2,3,6,7,8-HxCDD	15 (3.0)	5.0 (5.0–5.0)	8.0 (8.0–9.0)	12.2 (12.0–14.0)	23.0 (20.0–25.0)
1,2,3,7,8,9-HxCDD	345 (70.4)	<LOD	<LOD	2.0 (2.0–2.0)	4.0 (4.0–5.0)
1,2,3,4,6,7,8-HpCDD	3 (0.6)	5.5 (5.0–6.0)	7.0 (7.0–7.1)	10.0 (9.0–10.0)	17.0 (15.0–19.9)
OCDD	0 (0.0)	67.0 (64.0–70.0)	96.0 (91.0–100.0)	130.0 (130.0–150.0)	284.5 (241.2–320.0)
Total PCDDs (pg/g lipid)	–	82.0 (79.0–86.0)	120.0 (110.0–120.0)	160.0 (150.0–180.0)	320.0 (290.0–359.9)
Total PCDDs (pg TEQ/g lipid)	–	2.5 (2.4–2.6)	3.8 (3.6–4.0)	5.9 (5.3–6.2)	11.2 (9.9–12.7)
PCDFs (pg/g lipid)					
2,3,7,8-TeCDF	366 (74.6)	<LOD	<LOD	<LOD	2.0 (2.0–2.0)
1,2,3,7,8-PeCDF	398 (81.2)	<LOD	<LOD	<LOD	2.0 (1.0–2.0)
2,3,4,7,8-PeCDF	9 (1.8)	4.0 (4.0–4.0)	5.0 (5.0–5.0)	7.0 (7.0–7.5)	12.0 (11.0–13.9)
1,2,3,4,7,8-HxCDF	325 (66.3)	<LOD	< LOD	2.0 (2.0–2.0)	4.0 (3.0–5.0)
1,2,3,6,7,8-HxCDF	229 (46.7)	<LOD	2.0(<LOD–2.0)	3.0 (3.0–3.0)	5.0 (5.0–6.0)
1,2,3,7,8,9-HxCDF	490 (100.0)	<LOD	<LOD	<LOD	<LOD
2,3,4,6,7,8-HxCDF	460 (93.8)	<LOD	<LOD	<LOD	2.0 (<LOD–2.0)
1,2,3,4,6,7,8-HpCDF	374 (76.3)	<LOD	<LOD	<LOD	4.0 (3.0–5.0)
1,2,3,4,7,8,9-HpCDF	490 (100.0)	<LOD	<LOD	<LOD	<LOD
OCDF	490 (100.0)	<LOD	<LOD	<LOD	<LOD
Total PCDFs (pg/g lipid)	–	4.0 (4.0–4.0)	8.0 (7.0–9.0)	13.0 (12.0–14.0)	26.0 (23.1–30.0)
Total PCDFs (pg TEQ/g lipid)	–	1.2 (1.2–1.2)	1.8 (1.7–1.9)	2.6 (2.4–2.8)	4.7 (4.2–5.3)
Total (PCDDs+PCDFs) (pg/g lipid)	–	89.0 (85.0–96.0)	130.0 (120.0–131.9)	172.5 (160.0–190.0)	334.5 (300.0–379.9)
Total (PCDDs+PCDFs) (pg TEQ/g lipid)	–	3.8 (3.6–4.0)	5.7 (5.3–6.0)	8.3 (7.7–8.9)	16.0 (14.0–18.0)
Co-PCBs (non-ortho PCBs and mono-ortho PCBs)					
Non-ortho PCBs (pg/g lipid)					
3,3′,4,4’-TeCB (#77)	489 (99.8)	< LOD	< LOD	< LOD	< LOD
3,4,4′,5-TeCB (#81)	490 (100.0)	< LOD	< LOD	< LOD	< LOD
3,3′,4,4′,5-PeCB (#126)	56 (11.4)	20.0 (10.0–20.0)	20.0 (20.0–30.0)	40.0 (40.0–50.0)	90.0 (80.0–100.0)
3,3′,4,4′,5,5’-HxCB (#169)	28 (5.7)	20.0 (20.0–20.0)	25.0 (20.0–30.0)	40.0 (30.0–40.0)	74.5 (70.0–90.0)
Total non-ortho PCBs (pg/g lipid)	–	30.0 (30.0–40.0)	50.0 (50.0–50.0)	80.0 (70.0–90.0)	164.5 (141.2–180.0)
Total non-ortho PCBs (pg TEQ/g lipid)	–	2.1 (1.6–2.6)	3.2 (2.9–3.6)	5.2 (4.8–5.9)	11.4 (10.2–12.7)
2,3,3′,4,4’-PeCB (#105)	0 (0.0)	600.0 (544.3–650.0)	940.0 (890.0–1000.0)	1600.0 (1400.0–1756.2)	3400.0 (2900.0–3899.2)
Mono-ortho PCBs (pg/g lipid)					
2,3,4,4′,5-PeCB (#114)	0 (0.0)	180.0 (160.0–190.0)	290.0 (250.0–320.0)	480.0 (440.0–530.0)	1000.0 (881.2–1100.0)
2,3′,4,4′,5-PeCB (#118)	0 (0.0)	3175.0 (2900.0–3500.0)	5200.0 (4800.0–5600.0)	8400.0 (7800.0–9400.0)	18,000.0 (16,000.0–20,000.0)
2′,3,4,4′,5-PeCB (#123)	2 (0.4)	50.0 (40.0–50.0)	70.0 (70.0–80.0)	130.0 (120.0–140.0)	284.5 (231.2–349.9)
2,3,3′,4,4′,5-HxCB (#156)	0 (0.0)	1300.0 (1200.0–1400.0)	2100.0 (1980.4–2300.0)	3300.0 (3000.0–3600.0)	7045.0 (5912.0–8398.4)
2,3,3′,4,4′,5’-HxCB (#157)	0 (0.0)	350.0 (330.0–380.0)	570.0 (530.0–610.0)	880.0 (800.0–970.0)	1900.0 (1600.0–2200.0)
2,3′,4,4′,5,5’-HxCB (#167)	0 (0.0)	530.0 (480.0–570.0)	850.0 (790.0–940.0)	1300.0 (1200.0–1500.0)	3045.0 (2612.5–3300.0)
2,3,3′,4,4′,5,5’-HpCB (#189)	0 (0.0)	177.5 (160.0–190.0)	270.0 (250.0–290.0)	412.5 (390.0–450.0)	984.5 (833.7–1199.6)
Total mono-ortho PCBs (pg/g lipid)	–	6800.0 (6200.0–7200.0)	10,500.0 (9600.0–11,000.0)	16,250.0 (15,000.0–18,000.0)	34,450.0 (31,000.0–38,996.2)
Total mono-ortho PCBs (pg TEQ/g lipid)	–	0.2 (0.2–0.2)	0.3 (0.3–0.3)	0.5 (0.5–0.5)	1.0 (0.9–1.2)
Total Co-PCBs (pg/g lipid)	–	6800.0 (6200.0–7200.0)	11,000.0 (9700.0–12,000.0)	17,000.0 (15,000.0–18,000.0)	34,450.0 (31,124.9–38,996.2)
Total Co-PCBs (pg TEQ/g lipid)	–	2.4 (1.8–2.8)	3.6 (3.2–3.9)	5.7 (5.2–6.6)	12.0 (11.0–14.0)
Total (PCDDs + PCDFs + Co-PCBs) (pg/g lipid)	–	6915.0 (6267.1–7326.4)	11,057.0 (9848.7–11,857.8)	17,066.0 (15,202.8–18,191.6)	35,187.4 (31,280.6–39,107.0)
Total (PCDDs + PCDFs + Co-PCBs) (pg TEQ/g lipid)	–	6.1 (5.5–6.6)	9.4 (8.8–9.9)	14.0 (13.0–9.9)	27.5 (24.0–31.0)

95% confidence intervals are presented in parentheses

*SEDOCCH* survey on the exposure to dioxins and other chemical compounds in humans, *PCDDs* polychlorinated dibenzo-dioxins, *PCDFs* polychlorinated dibenzofurans, *Co-PCBs* coplanar polychlorinated biphenyls, *LOD* limit of detection

*A value of zero was assigned to concentrations below the LOD

**Fig. 2:**
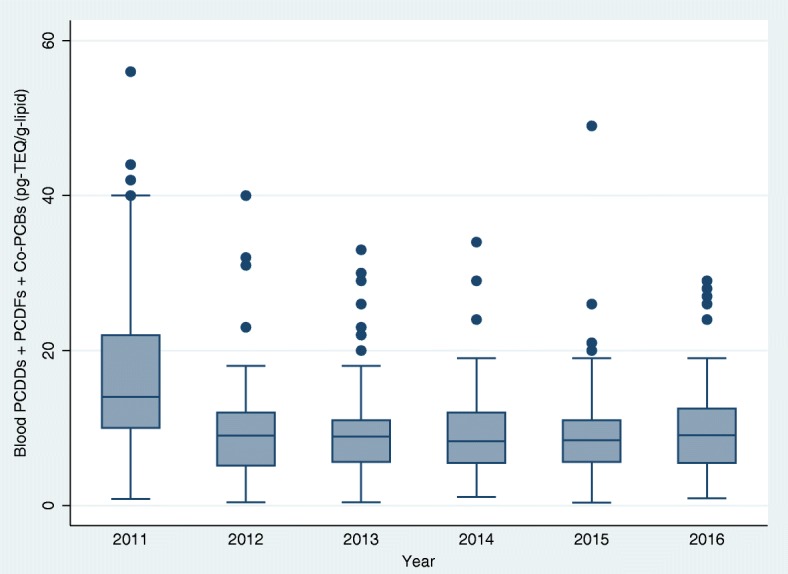
(Showing whiskers). Box plot (median, interquartile range and range) of total TEQ in the blood (in pg TEQ/g lipid) by survey year of the ‘survey on the exposure to dioxins and other chemical compounds in humans,’ 2011 to 2016. The median (range) of total TEQ by survey year was 14.0 (0.8–56.0), 9.0 (0.4–40.0), 8.9 (0.4–33.0), 8.3 (1.1–34.0), 8.4 (0.3–49.0) and 9.0 (0.9–29) in 2011, 2012, 2013, 2014, 2015 and 2016, respectively. The Kruskal-Wallis test (*p* < 0.001) was used to compare the differences in total TEQ in blood across the survey years. Definition of abbreviations: PCDDs, polychlorinated dibenzo-dioxins; PCDFs, polychlorinated dibenzofurans; Co-PCBs, coplanar polychlorinated biphenyls; TEQ, toxic equivalent

Figure 2 shows the blood dioxin trends by survey year. Overall, a significant decline in blood dioxins was observed from 2011 to 2016 (Kruskal-Wallis test, *p* < 0.001). As expected, we observed that blood dioxins increased with age (Additional file 2: Figure S1), with median blood dioxins being significantly lower (Mann-Whitney test, *p* < 0.001) in participants aged ≤ 39 years (5.4 pg TEQ/g lipid) than in those ≥ 40 (9.7 pg TEQ/g lipid). Of the 490 participants, 106 (21.6%) with a mean age of 55 years (range, 41–75) were found to have blood dioxins ≥ 16 pg TEQ/g lipid (median TEQ in the 2002–2010 SEDOCCH). The total blood TEQ medians (95% CI) were higher in areas in which the blood dioxin levels were reported to be high [10 (9.4–11) pg TEQ/g lipid] in the 2002–2010 SEDOCCH results (Wilcoxon rank-sum test, *p* < 0.001) compared with those where they were low [7.7 (6.9–8.7) pg TEQ/g lipid].

The median (95% CI) total TEQ in the blood of participants living in fishing villages [11.0 (9.7–12.0) pg TEQ/g lipid] was relatively higher compared to those in urban [7.4 (5.8–8.8) pg TEQ/g lipid] and agricultural/farming areas [8.3 (7.5–9.1) pg TEQ/g lipid]. The median (95% CI) total TEQ in the blood were significantly lower (Wilcoxon rank-sum test, *p* < 0.001) in urban participants [7.4 (5.8–8.8) pg TEQ/g lipid] than in rural participants [9.6 (9.1–10.0) pg TEQ/g lipid]. However, no significant blood dioxin differences were observed between the two smoking categories, with the median (95% CI) total TEQ in the blood of non-smokers and smokers being respectively 9.5 (8.8–10.0) pg TEQ/g lipid and 9.2 (7.9–10.0) pg TEQ/g lipid (Wilcoxon rank-sum test, *p* = 0.857).

### Estimated dioxin dietary intake

**Fig. 3:**
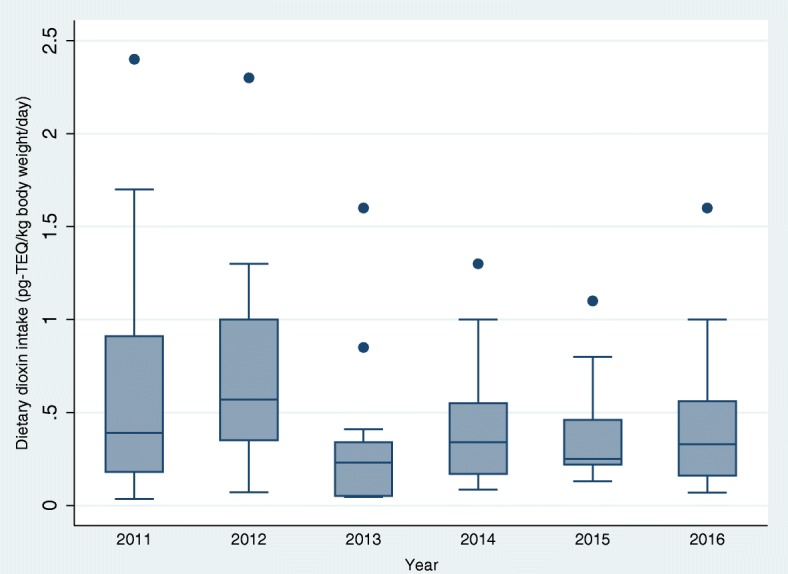
(Showing whiskers): Estimated dioxin dietary intake by year (*n* = 15/year). Box plot of total TEQ in food by survey year of the ‘survey on the exposure to dioxins and other chemical compounds in humans,’ 2011 to 2016 (*N* = 90) The median (range) of dioxin dietary intake (in pg TEQ/kg body weight/day) by survey year was 2011, 0.39 (0.03–2.4); 2012, 0.57 (0.07–2.3); 2013, 0.23 (0.04–1.6); 2014, 0.34 (0.08–1.3); 2015, 0.25 (0.13–1.1); and 2016, 0.33 (0.06–1.6). Kruskal-Wallis test: *χ*^2^ = 11.25; d.f. = 5; *p* = 0.046. TEQ, toxic equivalents.

**Fig. 4:**
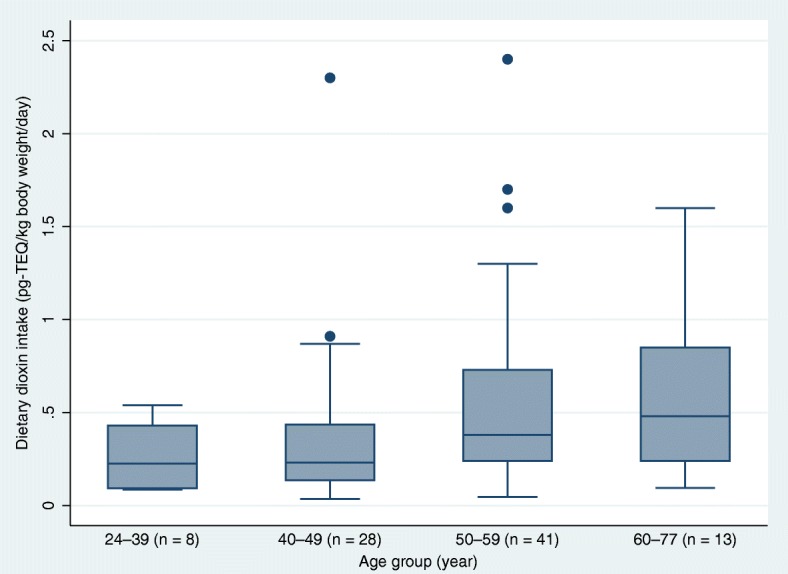
(Showing whiskers): Estimated dioxin dietary intake by age. Box plot of total TEQ in food by age group of the ‘survey on the exposure to dioxins and other chemical compounds in humans,’ 2011 to 2016 (N = 90). The median (range) of dioxin dietary intake (in pg TEQ/kg body weight/day) by age group (year) was 24–39, 0.22 (0.08–0.54); 40–49, 0.23 (0.03–2.3); 50–59, 0.38 (0.04–2.4); and 60–77, 0.48 (0.09–1.6). Kruskal-Wallis test: *χ*^2^ = 8.87; d.f. = 3; *p* = 0.031. TEQ, toxic equivalents.

Ninety samples were used to estimate the dioxin dietary intake. Overall, we observed a decline in the dioxin dietary intake per year (Fig. 3). The mean (standard deviation) dietary total TEQ intake was estimated at 0.49 (0.48) pg TEQ/kg body weight/day, and the median (95% CI) PCDDs/PCDFs and Co-PCBs and total TEQ intake were respectively, 0.16 (0.12–0.20), 0.16 (0.12–0.19) and 0.33 (0.25–0.40) pg/kg body weight/day (Additional file 3: Figure S2). The median (95% CI) TEQ dietary intake (pg/kg body weight/day) was found to be the highest in the fishing areas [0.35 (0.25–0.52)], followed by the urban areas [0.30 (0.09–1.07)] and the agricultural/farming areas [0.24 (0.19–0.45)]; however, the differences were not statistically significant (Kruskal-Wallis test, *p* = 0.411). The data also showed that total TEQ intake medians (95% CI) were similar in areas in which the blood dioxin levels were reported to be high [0.30 (0.24–0.38) pg/kg body weight/day] and low [0.39 (0.22–0.54) pg/kg body weight/day] in the 2002–2010 SEDOCCH results (Wilcoxon rank-sum test, *p* = 0.843). Total TEQ intake medians (range) were also similar (Wilcoxon rank-sum test, *p* = 0.739) between males [0.33 (0.35–2.30)] and females [0.32 (0.04–2.40)]. However, these medians tended to increase with age (Fig. 4).

### Relationship between blood dioxin TEQ and health parameters/clinical biomarkers

Table 3 shows the correlations between the blood total TEQ and health parameters/clinical biomarkers. Positive correlations were found between age and total TEQ in the blood, and there were also significant positive correlations between total TEQ in the blood and the estimated dietary TEQ intake, the BMI, abdominal circumferences, HbA1c, blood glucose, triglycerides, AST, ALT, GGT, EPA, and DHA. However, total TEQ in the blood had significant negative correlations with DGLA and weak negative correlations with triiodothyronine. Regarding the dietary dioxin intake, the results obtained without imputations of blood dioxin congeners showed that the Pearson’s correlation coefficients were similar to those with imputations. For instance, without imputations, and using the PMM and MICE, the correlations between total TEQ in the blood and the dietary dioxin intake had Pearson’s correlation coefficients of 0.371, 0.367 and 0.387, respectively.

**Table 3:** Pearson’s correlation between log-transformed blood dioxins (in pg TEQ/g lipid) and health outcomes, SEDOCCH 2011–2016

Variables	Total TEQ with ‘zero’ substituted for dioxin congeners < LOD		Total TEQ with dioxins imputed using PMM		Total TEQ with dioxins imputed using MICE	
	*r*	*p* value	*r*	*p* value	*r*	*p* value
Age (years)	0.443	< 0.001	0.556	< 0.001	0.501	< 0.001
BMI (kg/m^2^)	0.194	< 0.001	0.150	< 0.001	0.160	< 0.001
Abdominal circumference (cm)	0.164	0.001	0.142	0.001	0.139	0.001
Number of cigarettes smoked per day*	0.017	0.701	0.064	0.157	0.044	0.330
HbA1c (NGSP value, %)*	0.136	0.006	0.198	< 0.001	0.192	< 0.001
Blood glucose (mg/dL)	0.232	< 0.001	0.272	< 0.001	0.264	< 0.001
Insulin (μU/mL)	− 0.009	0.827	− 0.040	0.373	− 0.031	0.482
Triglycerides (mg/dL)	0.202	< 0.001	0.198	< 0.001	0.188	< 0.001
HDL cholesterol (mg/dL)	− 0.080	0.074	− 0.088	0.051	− 0.082	0.067
Free T3 (pg/mL)	− 0.099	0.028	− 0.098	0.029	− 0.095	0.034
Blood urea nitrogen (mg/dL)	0.093	0.038	0.114	0.011	0.112	0.013
Blood creatinine (mg/dL)^c^	− 0.030	0.497	− 0.046	0.309	− 0.040	0.366
AST (IU/L)	0.169	< 0.001	0.155	< 0.001	0.166	< 0.001
ALT (IU/L)	0.128	0.004	0.100	0.025	0.111	0.013
GGT (IU/L)	0.151	< 0.001	0.117	< 0.009	0.123	0.006
DGLA (μg/mL)	− 0.131	0.003	− 0.132	0.003	− 0.145	0.001
AA (μg/mL)	− 0.024	0.584	− 0.033	0.456	− 0.038	0.398
EPA (μg/mL)	0.452	< 0.001	0.473	< 0.001	0.463	< 0.001
DHA (μg/mL)	0.440	< 0.001	0.441	< 0.001	0.435	< 0.001
Dietary dioxin intake (pg TEQ/kg/day)*	0.371	< 0.001	0.367	< 0.001	0.387	< 0.001

All variables were log-transformed, except age and the number of cigarettes smoked per day

*SEDOCCH* survey on the exposure to dioxins and other chemical compounds in humans, *PCDDs* polychlorinated dibenzo-dioxins, *PCDFs* polychlorinated dibenzofurans, *Co-PCBs* coplanar polychlorinated biphenyls, *TEQ* toxicity equivalent, *BMI* body mass index, *HbA1c* haemoglobin A1c, *NGSP* National Glycohemoglobin Standardisation Programme, *HDL* high-density lipoprotein, *T3* triiodothyronine, *AST* aspartate aminotransferase, *ALT* alanine aminotransferase, *GGT* gamma-glutamyl transpeptidase, *DGLA* dihomo-gamma-linolenic acid, *AA* arachidonic acid, *EPA* eicosapentaenoic acid, *DHA* docosahexaenoic acid, *PMM* predictive mean matching, *MICE* multiple chained equations

*N = 490, except for HbA1c (*n* = 404), number of cigarettes smoked per day (*n* = 485) and dietary dioxin intake (*n* = 90). Missing data of covariates were not imputed

The results of the adjusted associations between the total blood dioxin TEQ and health parameters/clinical biomarkers are shown in Table 4. Consistent positive associations were found between the TEQ in the blood and age, BMIs, DHA and triglycerides while plasma DGLA was found to have a negative association. Using multiple imputation methods for the dioxin congeners concentrations below the LOD, the relationship between blood dioxins and health parameters/clinical biomarkers were found to be similar between the models that used imputed and non-imputed dioxin data. However, the adjusted *R*^2^ from the models that used the simple substitution dioxin data were different from the models that used dioxin data imputations, with the PMM (R^2^ = 0.480) and MICE (*R*^2^ = 0.440) providing stronger evidence compared with the simple substitution method (*R*^2^ = 0.410). In adjusted linear regression models that included smoking status, we found no association between smoking status and the total blood dioxin TEQ using the simple substitution method (beta = − 0.347; *p* = 0.420; *R*^2^ = 0.410), PMM (beta = 0.073; *p* = 0.856; *R*^2^ = 0.480), and MICE (beta = − 0.011; *p* = 0.777; *R*^2^ = 0.440). Including smoking status in the models had no effect on the significance of the models (Table 4) and increased slightly the variance inflation factor (VIF) from 1.33 to 1.42. It was also found that the VIF was 2.2 for EPA, DHA, DGLA, ALT, AST and GGT. However, VIF dropped to 1.1 when EPA, AST and GGT were excluded, indicating that there were some collinearities between clinical biomarkers [AST and GGT correlated with ALT (rho = 0.739 and rho = 0.624, respectively) whereas EPA with DHA (rho = 0.778)]. Using MICE, after multivariable adjustment, we also found associations between total blood dioxin TEQ and EPA (beta = 0.270; *p* < 0.001; *R*^2^ = 0.425). In the model restricted to the 90 participants for whom dietary dioxin intake estimations were available (listwise deletion), we could not detect an association between total blood dioxin TEQ and dietary dioxin intake after multivariable adjustment (Additional file 1: Table S2).

**Table 4:** Relationship between log-transformed total blood dioxins (in pg TEQ/g lipid) and health parameters/clinical biomarkers, 2011–2016 SEDOCCH (*N* = 490)

	Total TEQ with ‘zero’ substituted for dioxin congeners < LOD			Total TEQ with dioxins imputed using PMM			Total TEQ with dioxins imputed using MICE		
Predictor	Coefficient (*β*)	*p* value	Adjusted *R*^2^	Coefficient (*β*)	*p* value	Adjusted *R*^2^	Coefficient (*β*)	*p* value	Adjusted *R*^2^
			0.410			0.483			0.440
Age (years)	0.298	< 0.001		0.420	< 0.001		0.360	< 0.001	
BMI (kg/m^2^)	0.164	< 0.001		0.112	0.003		0.136	0.001	
DGLA (μg/mL)	− 0.380	< 0.001		− 0.360	< 0.001		− 0.376	< 0.001	
DHA (μg/mL)	0.362	< 0.001		0.315	< 0.001		0.321	< 0.001	
Triglycerides (mg/dL)	0.158	0.002		0.134	0.002		0.131	0.003	
Blood glucose (mg/dL)	0.057	0.142		0.098	0.008		0.087	0.024	
ALT (IU/L)	0.067	0.090		0.048	0.215		0.080	0.050	

Variables included in the model: age, BMI, triglycerides, blood glucose, blood urea nitrogen, ALT, DGLA, DHA, and sex

BMI, triglycerides, blood glucose, blood urea nitrogen, ALT, DGLA, and DHA were log-transformed

*SEDOCCH* survey on the exposure to dioxins and other chemical compounds in humans, *PMM* predictive mean matching, *MICE* multivariate imputation by chained equations, *BMI* body mass index, *ALT* alanine aminotransferase, *DGLA* dihomo-gamma-linolenic acid, *DHA* docosahexaenoic acid, *LOD* limit of detection

## Discussion

The SEDOCCH, which were designed to assess the health risks of pollutants such as dioxins and dietary dioxins intake in the general Japanese population, were a series of biomonitoring studies conducted by the MOEJ on non-occupationally exposed Japanese [18]. This study evaluated the progress made by the government to decrease the dioxin dietary intake and corresponding body burden.

### Current dioxin levels in Japanese from the 2011–2016 SEDOCCH data

It was found that the median concentration of blood dioxin TEQ in the general adult Japanese population was 9.40 pg TEQ/g lipid and the dietary dioxin intake was 0.33 pg TEQ/kg body weight/day. Blood dioxins (5.4 pg TEQ/g lipid) in participants aged ≤ 39 years were particularly striking, suggesting that dioxin exposure intensity and accumulation time were probably limited in this age group. However, only a small percentage (7.3%) of the participants aged ≤ 39 years was included in this study. Higher median blood dioxins were found in participants aged ≥ 40 years old, which supported findings in previous studies on the lipophilicity of dioxins and their accumulation in adipose tissues over a lifetime [29]. The national estimates of blood dioxins found in this study matched international trends and were in line with a review that reported worldwide decreasing PCDD/PCDFs TEQ values in samples collected up to 2008 [30], but were lower than the median values reported in Russian women in 2010 [31]. TEQ values or that of congeners reported in this study might be difficult to compare between countries/studies because there is significant heterogeneity in the study designs, analytical methods, handling values below the LOD and TEF utilisation. For instance, a study conducted in rural Germany reported median blood dioxins of 7.5 pg TEQ/g lipid (vs. 9.4 pg TEQ/g lipid in this study) [32]. However, our study population differed considerably from the study conducted in Germany. The latter study used the 2005 WHO TEFs (vs. 2006 WHO TEFs in this study) with a sample size of 70 (vs. 490 in this study) and its participants’ age ranged from 4 to 76 years (vs. 24–77 years in this study). The fact that the German’s study included relatively younger participants might have contributed to the relatively lower reported total TEQ value. The PCDD/PCDFs and Co-PCBs values found in this study are similar to those reported in another recent study conducted in Germany that had looked at chlorinated dioxins in 42 blood donors [33]. The study in the German blood donors has documented a PCDD/PCDFs value of 6.2 pg TEQ/g lipid (vs. 5.7 pg TEQ/g lipid in this study) and Co-PCBs of 4.1 pg TEQ/g lipid (vs. 3.6 pg TEQ/g lipid) among participants aged 20 to 68 years [33]. Moreover, a study conducted in Ghana among individuals working at e-waste recycling sites and controls who were not directly exposed to e-waste recycling activities reported a PCDD/PCDFs value of 4.6 pg TEQ/g lipid (vs. 5.7 pg TEQ/g lipid in this study) in controls with a mean age of 24.4 years (vs. 49.4 years in this study) [34].

In this study, the dietary dioxin exposure was estimated using a duplicate diet analysis from three consecutive days [15], from which it was found that none of the participants exceeded the tolerable daily dioxin intake of 4 pg/kg body weight/day stipulated in the ‘Act on Special Measures against Dioxins’ [11]. The highest dietary dioxins intake was found in participants living in fishing areas (0.35 pg/kg body weight/day), which supported previous data that suggested that fish and sea foods were more vulnerable to dioxin contamination [8, 35]. These findings were also consistent with a report by Arisawa et al., in which it was found that the highest dietary dioxin intake was in Japanese from fishing villages; however, the previously reported value (0.98 pg/kg body weight/day) was higher than that found in the current study [15]. The estimated mean dietary dioxin intake (0.49 pg TEQ/kg body weight/day) found in this study was approximately three times lower that that reported in a French study from 2015, which reported a mean of 1.3 pg TEQ/kg body weight/day [36]. However, in the latter study, the data were collected between 1993 and 2008 when dioxin levels in the environment were probably higher than now.

### Comparison of the 2002–2010 and 2011–2016 SEDOCCH data

In reference to age, it was expected that the dioxin levels would be higher in participants in this study (mean age = 49.9 years) than those in the 2002–2010 SEDOCCH (mean age = 44.5 years; *N* = 2264) [20]; however, this was not found. A comparison of the median blood dioxin levels reported in this study found that the blood dioxin TEQ were 41.3% lower than that those reported in the 2002–2010 SEDOCCH, which had reported a median of 16 pg TEQ/g lipid. The dietary dioxin intake was also found to be 41.1% lower in the 2011–2016 SEDOCCH than the median 0.56 pg TEQ/kg body weight/day reported in the 2002–2010 SEDOCCH (*N* = 625) [37], providing evidence that the blood dioxin and dietary dioxin intake levels were decreasing nationally. The blood dioxin and dietary dioxin intake were also found to have decreased in a similar way (approximately 41%), which was in line with previous findings that documented significant declines in dioxin levels in Japan [12]. A typical dioxin congener, 2,3,7,8-tetrachlorodibenzo-para- dioxin (TCDD), has a biological half-life of 6 to 11 years in adults [38, 39], and all other congeners have similar half-lives. In this study, 77.1% of the blood samples had undetectable TCDD concentrations (Table 2). The TCDD level found in this study is lower compared with that in a previous study in Italy that has found a median of 0.68 pg TEQ/g lipid [40]. Previous studies have identified diet as a major source of dioxin exposure [8, 35]; however, dioxin elimination can be influenced by several factors such as body fat, which has been found to increase/decrease blood dioxin levels and elimination rates [38]. Since no significant differences were found in the amount of body fat between the 2002–2010 SEDOCCH (BMI ≈ 23 kg/m^2^) [20] and the 2011–2016 SEDOCCH (BMI ≈ 23 kg/m^2^), it was surmised that the decrease in dietary dioxin intake had contributed to the decline in blood dioxins. Studies have shown that food dioxins come mainly from atmospheric deposition [41]; therefore, the government’s efforts to reduce dioxin emissions may have played a significant role in decreasing the dioxin levels in Japan.

Likewise, the upper blood dioxins range reported in this study (56 pg TEQ/g lipid) fell well below the upper range of 130 pg TEQ/g lipid [37] estimated in the 2002–2010 SEDOCCH, providing additional evidence that the blood dioxins in the Japanese population may be decreasing. Although other explanations might be possible, one hypothesis was that this decreasing trend could have been due to the decline of dioxins in the atmosphere because of the strict Japanese government’s laws that limit incineration emissions. Even though it was found that the blood dioxins and dietary dioxin intake were declining in the Japanese population sample, they should not be interpreted as meaning that people now have a zero risk of dioxin exposure [15].

In this study, 21.6% of the participants still had blood dioxins ≥ 16 pg TEQ/g lipid, which was the median blood dioxin TEQ reported in the 2002–2010 SEDOCCH [18, 37]. The higher blood dioxin levels reported in the 2002–2010 SEDOCCH were found to be linked to adverse health outcomes [2, 3, 8, 14–20]. Therefore, the blood dioxins of ≥ 16 pg TEQ/g lipid found in this study warrant further investigation because of the potential long-term health effects. These results also have implications for the MOEJ and highlighted the critical need for longitudinal monitoring to assess long-term public exposure to dioxins and to reduce background exposure in the general population.

### Relationship between blood dioxins and health parameters/clinical biomarkers

The correlations reported in the 2002–2010 SEDOCCH between blood dioxins and health parameters [2] were also found in this study (2011–2016 SEDOCCH) for data based both on non-detects equal to zero and the imputed data (Table 3). In particular, participants with higher blood dioxin levels were more likely to have higher BMIs, more elevated triglycerides, higher DHA and EPA, and lower DGLA (Table 4) and then could be explained by age, but not by smoking status. The associations between age and blood dioxins found in the present study were expected, as chlorinated dioxins are known to be generally more resistant to biotransformation and elimination from the body. It was assumed that the total body blood dioxins burden in this group was probably due to the cumulative lifetime exposure to dioxins rather than because of recent exposures, as the dioxins half-life in the human body can exceed 7 years. Therefore, the results might have reflected the dioxins the participant had accumulated over a long time and a slower dioxin elimination [42].

Our findings that biomarkers of fish intake, DHA and EPA were associated with blood dioxins are in line with the view of marine fish intake as one of the foods that influence blood dioxins in Japan [14]. They support the hypothesis that a significant portion of blood total TEQ in Japan is from fish intake. The association between blood dioxins and fish had been reported in the existing literature [43–45]. Yet a study conducted in Russia could not find associations between fish intake and blood dioxins in the overall participants though some participants who consumed high quantities of fish showed higher serum dioxin levels [31]. While smoking status has been found to be a predictor of blood dioxins [38, 41, 46], the findings that smoking status was not a significant predictor was unexpected. Blood dioxin levels were similar between never smokers and current smokers. The relatively small number (21%, 102/490) of current smokers and their relatively low mean age (compared with never smokers) in this study could be the most direct explanation for these unexpected findings. Alternatively, this lack of association between blood dioxins and smoking could mean that the dioxin levels were low in tobacco smoked by these participants.

However, the associations previously reported in Japan between blood dioxins and dietary dioxin intake [15, 16] were not found in the complete case analyses (i.e. when the models were restricted to the 90 participants for whom dietary dioxin intake estimations were available). The non-detection of most congeners with high TEF such as TCDD and tetrachlorodibenzofuran (TCDF) in blood samples along with the non-representativeness of the study population in the participant sub-sample and the relatively small sample size after the list wise deletion (*n* = 60 and *n* = 30 for high and low dioxin level areas, respectively) could possibly explain these lack of associations (Additional file 1: Table S2). In contrast, when dietary dioxin intake was removed from the models, with or without the imputed dioxin data, associations were found between blood dioxins and some health parameters/clinical biomarkers (Table 4). The results of multiple regression analysis for associations between blood dioxin levels and food dioxin intake showed that the effect of diet was small. One possible interpretation of the null relationship is these participants’ overall low exposure. To investigate the accurate kinetics, it is necessary that research combines the exposure and the pharmacokinetics models. In the present study, the mean dioxin dietary intake level was found to be within tolerable daily intake levels and was not found to be associated with blood dioxins after adjusting for potential confounders. Although higher dioxin levels have been reported in the Japanese, in addition to dioxin resistance to degradation, this finding suggested that there was a low accumulation of dioxins in the food consumed by the study sample. It was concluded that these results reflected the efforts made by the Japanese government to reduce environmental dioxins through initiatives such as proper waste incineration and the cleaning of dioxin-contaminated soil [12]. It is also possible that the dioxin intake in this study was underestimated as they were only estimated in 90 participants from food samples that were collected for only 3 days. Nevertheless, DHA and EPA were used to account for the foods that may influence blood dioxins among the Japanese. These findings add to the literature about the appropriate handling of missing data in epidemiological investigations [47–49]. The simple substitution of the dioxin congener values below the LOD is likely to generate biased results (Table 4) because the simple substitution methods caused an absence of any associations between the blood dioxins and the blood glucose. Therefore, the use of MICE and PMM to account for the missing data among the dioxin congeners was able to yield adequate results and reduce the possibility of making erroneous conclusions. Although MICE and PMM are based on different algorithms, in this study, they appeared to be similar regarding robustness.

### Limitations of the study

Although this study considered all necessary factors, there were some potential limitations. First, there was possibly some bias in the participants’ enrolment; specifically, participation was voluntary, and therefore, the participants were not randomly enrolled. The 2011 to 2016 sampling was primarily concentrated in areas where dioxin levels had been found to be high or low in the 2002–2010 SEDOCCH survey [16]. Therefore, the selection of the participants in the 2011–2016 SEDOCCH may not have been truly represented the Japanese population. Therefore, the 2011–2016 SEDOCCH sample size may not have been large enough to truly represent the Japanese population. However, this limitation was mitigated by recruitment in all five Japanese regions and the target number of participants recruited each year. SEDOCCH investigators are now working to improve the next SEDOCCH to ensure a true representativeness of the Japanese population. Second, although the data on the individuals invited to participate in the study and the response rates were not available, the study succeeded in recruiting the planned number of participants for the study design. Even though the interviews were conducted face-to-face, the absence of a bias influence on the quality of responses obtained cannot be ruled out [50]. Third, the cross-sectional design of the study limited the interpretation of the findings, especially when the half-life of dioxins is known to exceed 7 years [4]. As most participants were only sampled once, it was difficult to estimate the dioxin levels over time along with the rates of decay. Previous studies have reported that dioxin elimination can occur faster for people with higher dioxin levels [42]. In this study, 63.5% of the participants had been recruited in areas where the dioxin levels were reported as high (median 21.5 to 40.9 pg TEQ/g lipid) in the 2002–2010 SEDOCCH results; therefore, it could be speculated that the participant group may have experienced faster dioxin elimination. This observation and the low dioxin intake might be the reason for the dramatic decline in dioxins, even though dioxins tend to have longer half-lives. The oversampling of participants from prefectures in which the blood dioxin levels had been found to be high in the 2002–2010 SEDOCCH surveys contributed to enabling the evaluation of the government action to reduce dioxins in Japan. In these areas, the median blood dioxins were 10 pg TEQ/g lipid in the 2011–2016 SEDOCCH surveys.

Fourth, comparing our dietary dioxin intake measurements to the values reported in the 2002–2010 SEDOCCH surveys may also have introduced some bias. This is for reasons that the 2002–2010 SEDOCCH dietary data are more than 10 years old and were carried out in participants who were likely to live in urban areas (vs. rural areas in this study). However, the 2002–2010 SEDOCCH dietary dioxin intake estimations were from nationwide surveys and were set as a reference by the MOEJ for this report.

Despite these limitations, this study contributes to studies on dioxin levels. First, the findings provide insights into planning future epidemiological research to address dioxin-related issues and assists in public-policy decisions. Second, missing data is uncommon; for example, the questionnaire answers only had a little missing data because individual interviews were carried to accompany the previously completed self-administered questionnaire.

## Conclusions

Median blood dioxins and dietary dioxin intake were found to be approximately 41% lower in this study compared with those in the 2002–2010 SEDOCCH. It was found that the measures and actions undertaken in Japan have possibly contributed to these reductions in the body burden of dioxins in the Japanese population. In our non-occupationally exposed Japanese population, blood dioxins were found to be associated with clinical biomarkers including DHA and EPA. As fish intake may benefit health in humans, we recommend additional government actions to further reduce potential dioxin contaminants in fish.

## Additional files

Additional file1: **Table S1.** Health parameters/clinical biomarkers of 490 participants in SEDOCCH 2011–2016. **Table S2.** Linear regression models of log-transformed blood dioxins (in pg TEQ/g lipid) and dietary dioxin intake (pg TEQ/kg/day), 2011–2016 SEDOCCH. (DOCX 1923 kb)

Additional file 2: **Figure S1.** Box plot of total TEQ in the blood (in pg TEQ/g lipid) by age group of the ‘survey on the exposure to dioxins and other chemical compounds in humans,’ 2011 to 2016 (*N* = 490). The median (range) of total TEQ by age group (year) was 24–39: 5.4 (0.7–13.0); 40–49: 7.8 (0.4–34.0); 50–59: 12.0 (0.4–56.0); and 60–77: 15 (1.9–44.0). Kruskal-Wallis test: *χ*^2^ = 93.47; d.f. = 3; *p* < 0.001. TEQ, toxic equivalents. (PDF 231 kb)

Additional file 3: **Figure S2.** Estimated dioxin dietary intake by areas. Box plot of total TEQ in food (in pg TEQ/kg body weight/day) by survey area of the ‘survey on the exposure to dioxins and other chemical compounds in humans,’ 2011 to 2016 (*N* = 90). This figure shows the estimated median (range) dioxin intake for urban areas [PCDDs/PCDFs = 0.15 (0.04–0.51); Co-PCBs = 0.15 (0.03–1.0); dietary total TEQ intake = 0.31 (0.07–1.30)], farming areas [PCDDs/PCDFs = 0.12 (0.02–0.58); Co-PCBs = 0.12 (0.02–1.10); dietary total TEQ intake = 0.24 (0.04–1.60)], and fishing areas [PCDDs/PCDFs = 0.16 (0.02–1.0); Co-PCBs = 0.17 (0.02–1.80); dietary total TEQ intake = 0.35 (0.05–2.40)]. Definition of abbreviations: PCDDs, polychlorinated dibenzo-dioxins; PCDFs, polychlorinated dibenzofurans; Co-PCBs, coplanar polychlorinated biphenyls; TEQ, toxic equivalents. (PDF 237 kb)

## Abbreviations

ALT: Alanine aminotransferase
AST: Aspartate aminotransferase
BMI: Body mass index
CI: Confidence interval
Co-PCBs: Coplanar polychlorinated biphenyls
DGLA: Dihomo-gamma-linolenic acid
DHA: Docosahexaenoic acid
EDTA-2NA: Ethylenediaminetetraacetic acid disodium salt
EPA: Eicosapentaenoic acid
GGT: Gamma-glutamyl transpeptidase
HbA1c: Haemoglobin A1c
HDL: High-density lipoprotein
LOD: Limit of detection
MICE: Multiple imputations by chained equations
MOEJ: The Ministry of the Environment of Japan
NIES: National Institute for Environmental Studies
PCDDs: Polychlorinated dibenzo-p-dioxins
PCDFs: Polychlorinated dibenzofurans
PMM: Predictive mean matching
SEDOCCH: Survey on the exposure to dioxins and other chemical compounds in humans
T3: Triiodothyronine
T4: Thyroxine
TCDD: Tetrachlorodibenzo-para- dioxin
TCDF: Tetrachlorodibenzofuran
TEFs: Toxic equivalence factors
TEQ: Toxic equivalent
VIF: Variance inflation factor
WHO: World Health Organization
