# Supplementary material for: Dioxins levels in human blood after implementation of measures against dioxin exposure in Japan
Source: Environ Health Prev Med. 2019 Jan 10;24:6. doi: 10.1186/s12199-018-0755-7 (PMC6329082; doi:10.1186/s12199-018-0755-7)
Supplement: Supplementary file 1 — Table S1. Health parameters/clinical biomarkers of 490 participants in SEDOCCH 2011–2016. Table S2. Linear regression models of log-transformed blood dioxins (in pg TEQ/g lipid) and dietary dioxin intake (pg TEQ/kg/day), 2011–2016 SEDOCCH. (DOCX 1923 kb) [file 12199_2018_755_MOESM1_ESM.docx]

Dioxins levels in human blood after implementation of measures against dioxins exposure in Japan

Basilua Andre MUZEMBO^1, 5^ [muzembo_andre@iuhw.ac.jpl](mailto:muzembo_andre@iuhw.ac.jpl); Miyuki IWAI-SHIMADA^1^ [iwai.miyuki@nies.go.jp](mailto:iwai.miyuki@nies.go.jp); Tomohiko ISOBE^1^ [isobe.tomohiko@nies.go.jp](mailto:isobe.tomohiko@nies.go.jp); Kokichi ARISAWA^2^ [karisawa@tokushima-u.ac.jp](mailto:karisawa@tokushima-u.ac.jp); Masayuki SHIMA^3^ [shima-m@hyo-med.ac.jp](mailto:shima-m@hyo-med.ac.jp); Tetsuhito FUKUSHIMA^4^ [t-fuku@fmu.ac.jp](mailto:t-fuku@fmu.ac.jp); Shoji F. NAKAYAMA^1*^ +81-29-850-2786 fabre@nies.go.jp

^1^ Centre for Health and Environmental Risk Research
National Institute for Environmental Studies
Tsukuba 305-8506, Japan

^2^ Institute of Biomedical Sciences, Tokushima University Graduate School
Tokushima 770-8503, Japan

^3^ Department of Public Health
Hyogo College of Medicine
Hyogo 663-8501, Japan

^4^ Department of Hygiene and Preventive Medicine
School of Medicine, Fukushima Medical University
Fukushima 960-1295, Japan

^5^Department of Public Health, School of Medicine
International University of Health and Welfare
Narita, Japan

Table S1: Health parameters/clinical biomarkers of 490 participants in SEDOCCH 2011–2016

| Characteristics | Male (N = 242) | Female (N = 248) | All (N = 490) | *p-*value^§^ |
| --- | --- | --- | --- | --- |
|  | n (%), or mean (STD), or median (IQR) | n (%), or mean (STD), or median (IQR) | n (%), or mean (STD), or median (IQR) |  |
| Height (cm)^a^ | 170.0 ± 6.1 | 157.5 ± 5.8 | 163.7 ± 8.5 | <0.001 |
| Weight (kg)^a^ | 70.8 ± 11.4 | 57.1 ± 9.6 | 63.9 ± 12.5 | <0.001 |
| Abdominal circumference (cm)^a^ | 86.4 ± 8.6 | 81.3 ± 9.7 | 83.8 ± 9.5 | <0.001 |
|  |  |  |  |  |
| Systolic BP (mmHg)^b^ | 135.6 ± 17.9 | 131.2 ± 21.6 | 133.4 ± 20.0 | 0.007 |
| Diastolic BP (mmHg)^b^ | 84.4 ± 12.1 | 80.1 ± 13.1 | 82.2 ± 12.8 | <0.001 |
|  |  |  |  |  |
| HbA1c (NGSP value, %)^c^ |  |  |  | 0.513 |
| <5.6 | 127 (25.9) | 135 (27.5) | 262 (53.4) |  |
| ≥5.6 | 64 (13.0) | 78 (15.9) | 142 (28.9) |  |
| Unknown/missing | 51 (10.4) | 35 (7.1) | 86 (17.5) |  |
|  |  |  |  |  |
| Blood glucose (mg/dL)^d^ | 90 (86, 9) | 88 (82, 9) | 86 (84, 9) | <0.001 |
|  |  |  |  |  |
| Self-reported diabetes^c^ |  |  |  | 0.069 |
| No | 220 (44.9) | 235 (47.9) | 455 (92.8) |  |
| Yes | 22 (4.4) | 13 (2.6) | 35 (7.1) |  |
|  |  |  |  |  |
| Blood urea nitrogen (mg/dL)^d^ | 13 (11, 16) | 12 (10, 14) | 12 (10, 15) | <0.001 |
|  |  |  |  |  |
| Blood creatinine (mg/dL)^d^ | 0.77 (0.6, 0.8) | 0.5 (0.5, 0.6) | 0.6 (0.5, 0.7) | <0.001 |
|  |  |  |  |  |
| AST (IU/L)^d^ | 22 (18, 27) | 19 (17, 23) | 20 (18, 25) | <0.001 |
| ALT (IU/L)^d^ | 23 (17, 34) | 16 (12, 21) | 19 (14, 28) | <0.001 |
| GGT (IU/L)^d^ | 37 (24, 69) | 18 (14, 28) | 25 (17, 45) | <0.001 |
|  |  |  |  |  |
| DGLA (µg/mL)^d^ | 37.2 (28.0, 48.5) | 36.6 (27.4, 47.9) | 37.1 (27.8, 48.1) | 0.373 |
| AA (µg/mL)^d^ | 173.1 (141.5, 206.8) | 166.6 (134.7, 208.3) | 170.1 (139.1, 207.4) | 0.214 |
| EPA (µg/mL)^d^ | 43.4 (27.7, 76.6) | 44.7 (27.9, 67.0) | 43.9 (71.0, 27.0) | 0.464 |
| DHA (µg/mL)^d^ | 122 (86.8, 157.3) | 122.4 (94.7, 160.4) | 122.0 (91.6, 159.0) | 0.430 |
|  |  |  |  |  |
| Triglycerides (mg/dL)^d^ | 102 (72, 171) | 79 (58, 111) | 87 (63, 132) | <0.0001 |
| HDL cholesterol (mg/dL)^d^ | 56 (47, 64) | 66 (57, 77) | 60 (51, 73) | <0.0001 |
|  |  |  |  |  |
| Free T3 (pg/mL)^d^ | 3.4 (3.4, 3.6) | 3.1 (3.3, 2.9) | 3.3 (3.0, 3.5) | <0.0001 |
| Free T4 (ng/dL)^d^ | 1.3 (1.2, 1.4) | 1.2 (1.1, 1.3) | 1.2 (1.1, 1.3) | <0.0001 |

Definition of abbreviations: SEDOCCH, survey on the exposure to dioxins and other chemical compounds in humans; STD, standard deviation; IQR, interquartile range; BP, blood pressure; HbA1c, haemoglobin A1c; NGSP, national glycohaemoglobin standardisation programme; HDL, high-density lipoprotein; T3, triiodothyronine; T4, thyroxine; AST, aspartate aminotransferase; ALT, alanine aminotransferase; GGT, gamma-glutamyl transpeptidase; DGLA, dihomo-gamma-linolenic acid; AA, arachidonic acid; EPA, eicosapentaenoic acid; DHA, docosahexaenoic acid.

^a^Mean ± standard deviation;

^b^Mean (range);

^c^N (%);

^d^Median (25%, 75%). ^§^P-values are comparing male and female participants.

Data were missing for blood pressure (n = 1) and HbA1c (n = 86).

Table S2: Linear regression models of log-transformed blood dioxins (in pg-TEQ/g-lipid) and dietary dioxins intake (pg-TEQ/kg/day), 2011–2016 SEDOCCH^†^

|  | Overall (N= 90) | | |
| --- | --- | --- | --- |
| Models | Coefficient (β) | p-value | Adjusted R^2^ |
| Total TEQ with “zero” substituted for dioxins congeners <LOD | 0.111 | 0.180 | 0.530 |
| Total TEQ with dioxins imputed using PMM | 0.093 | 0.251 | 0.544 |
| Total TEQ with dioxins imputed using MICE | 0.116 | 0.167 | 0.517 |

Variables included in the model: Age, BMI, blood glucose, blood urea nitrogen, ALT, DGLA, DHA, and sex.

BMI, blood glucose, blood urea nitrogen, ALT, DGLA, and DHA were log-transformed.

Definition of abbreviations: SEDOCCH, survey on the exposure to dioxins and other chemical compounds in humans; BMI, body mass index; ALT, alanine aminotransferase; DGLA, dihomo-gamma-linolenic acid; and DHA, docosahexaenoic acid; PMM, predictive mean matching; MICE, multivariate imputation by chained equations; LOD, limit of detection.

^†^The analyses were restricted to participants for whom dietary dioxins intake data were available.
