# Supplementary figures and images for: Dioxins levels in human blood after implementation of measures against dioxin exposure in Japan
Source: Environ Health Prev Med. 2019 Jan 10;24:6. doi: 10.1186/s12199-018-0755-7 (PMC6329082; doi:10.1186/s12199-018-0755-7)

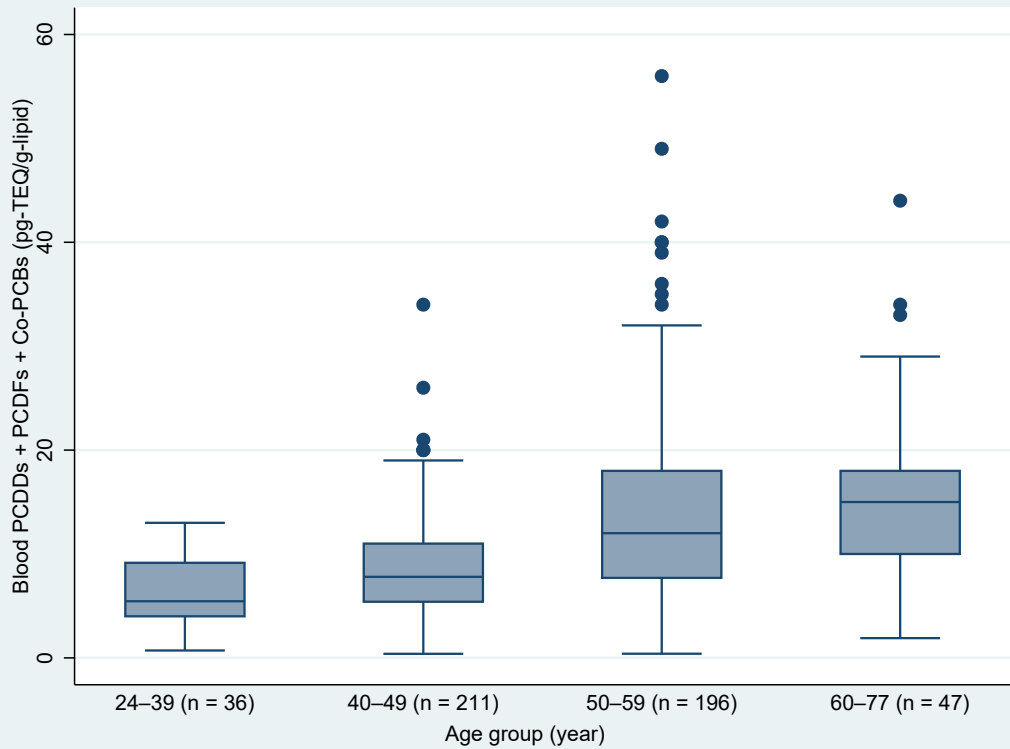

Supplement: Supplementary file 2 — Figure S1. Box plot of total TEQ in the blood (in pg TEQ/g lipid) by age group of the ‘survey on the exposure to dioxins and other chemical compounds in humans,’ 2011 to 2016 (N = 490). The median (range) of total TEQ by age group (year) was 24–39: 5.4 (0.7–13.0); 40–49: 7.8 (0.4–34.0); 50–59: 12.0 (0.4–56.0); and 60–77: 15 (1.9–44.0). Kruskal-Wallis test: χ2 = 93.47; d.f. = 3; p < 0.001. TEQ, toxic equivalents. (PDF 231 kb) [file 12199_2018_755_MOESM2_ESM.pdf]

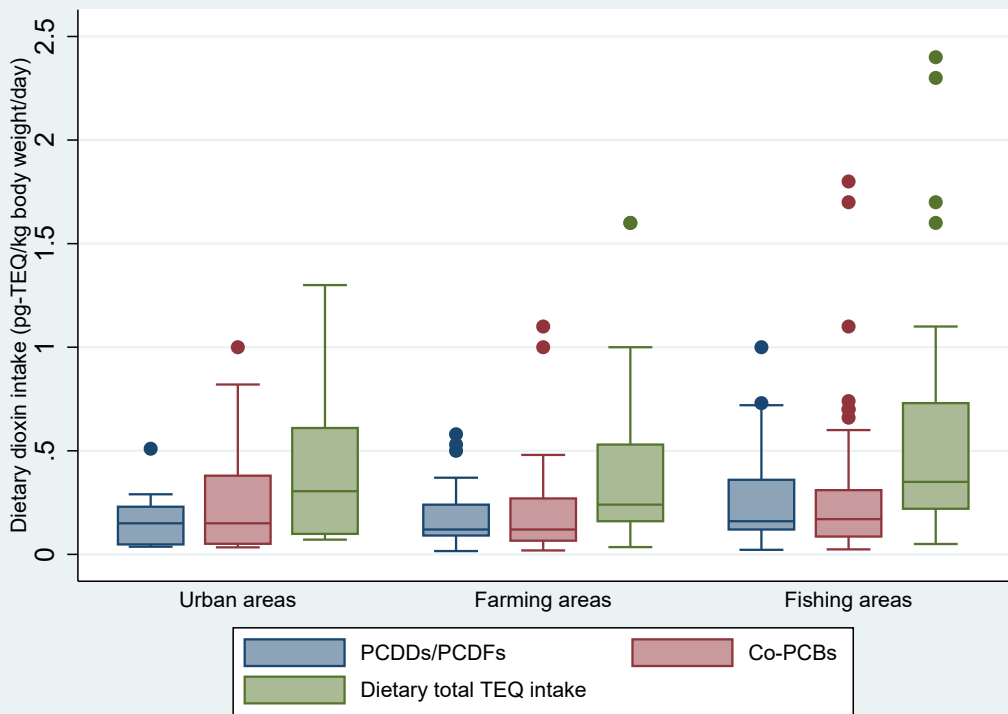

Supplement: Supplementary file 3 — Figure S2. Estimated dioxin dietary intake by areas. Box plot of total TEQ in food (in pg TEQ/kg body weight/day) by survey area of the ‘survey on the exposure to dioxins and other chemical compounds in humans,’ 2011 to 2016 (N = 90). This figure shows the estimated median (range) dioxin intake for urban areas [PCDDs/PCDFs = 0.15 (0.04–0.51); Co-PCBs = 0.15 (0.03–1.0); dietary total TEQ intake = 0.31 (0.07–1.30)], farming areas [PCDDs/PCDFs = 0.12 (0.02–0.58); Co-PCBs = 0.12 (0.02–1.10); dietary total TEQ intake = 0.24 (0.04–1.60)], and fishing areas [PCDDs/PCDFs = 0.16 (0.02–1.0); Co-PCBs = 0.17 (0.02–1.80); dietary total TEQ intake = 0.35 (0.05–2.40)]. Definition of abbreviations: PCDDs, polychlorinated dibenzo-dioxins; PCDFs, polychlorinated dibenzofurans; Co-PCBs, coplanar polychlorinated biphenyls; TEQ, toxic equivalents. (PDF 237 kb) [file 12199_2018_755_MOESM3_ESM.pdf]
